# Weight Gain Is Associated with Short-Term FIB-4 Increase and Concurrent Metabolic Change in People with HIV and Metabolic Dysfunction-Associated Steatotic Liver Disease

**DOI:** 10.3390/v18080896

**Published:** 2026-08-14

**Authors:** Wei Xu, Li Liu, Meiyan Sun, Renfang Zhang, Jun Chen, Yinzhong Shen

**Affiliations:** Department of Infection and Immunity, Shanghai Public Health Clinical Center, Fudan University, Shanghai 201508, China; 24111300008@m.fudan.edu.cn (W.X.); liuli@shphc.org.cn (L.L.); sunmeiyan@shphc.org.cn (M.S.)

**Keywords:** HIV, metabolic dysfunction-associated steatotic liver disease, FIB-4, longitudinal change, weight gain, obesity

## Abstract

Background: Short-term variation in the Fibrosis-4 index (FIB-4) may identify people who warrant further liver assessment. We evaluated longitudinal FIB-4 changes and associated metabolic factors in people with HIV (PWH) and metabolic dysfunction-associated steatotic liver disease (MASLD). Methods: This retrospective cohort included 683 PWH with ultrasound-defined MASLD who underwent routine clinical and laboratory assessments approximately every three months. A prespecified FIB-4 increase required both a ≥20% rise and crossing to ≥1.3 among participants with baseline FIB-4 < 1.3, or a ≥20% rise among those with baseline FIB-4 ≥ 1.3. Results: Of 683 enrolled participants, 655 were analyzed longitudinally. Over a median follow-up of 12.0 months, 59 (9.0%) met the FIB-4 increase definition, corresponding to 8.8 events per 100 person-years. Weight gain was associated with this outcome (adjusted hazard ratio 1.19 per 1% increase, 95% CI 1.14–1.24), whereas HIV- and antiretroviral therapy-related variables were not. Transition from non-obesity to obesity was associated with the outcome (odds ratio 8.45, 95% CI 3.57–20.02). Weight change also correlated with concurrent changes in total cholesterol, triglycerides, fasting glucose, and liver enzymes (all *p* < 0.01). Conclusions: Weight gain was associated with short-term FIB-4 increase and with concurrent metabolic change in PWH with MASLD.

## 1. Introduction

By the end of 2024, an estimated 40.8 million people worldwide were living with HIV, and 31.6 million (77%) were receiving antiretroviral therapy (ART) [1]. In China, approximately 1.329 million people were living with HIV by mid-2024 [2]. As survival has improved, metabolic comorbidity and liver disease have become increasingly important in people with HIV (PWH). Metabolic dysfunction-associated steatotic liver disease (MASLD) affects approximately 26–40% of PWH and contributes to hepatic and extrahepatic morbidity [3,4,5,6].

Clinical guidelines recommend non-invasive tests to identify patients who may need further assessment for advanced fibrosis [7,8,9]. The Fibrosis-4 index (FIB-4) is inexpensive and widely available, but it is calculated from age, aspartate aminotransferase (AST), alanine aminotransferase (ALT), and platelet count. These components can change because of intercurrent illness or metabolic and laboratory fluctuations. A threshold of 1.3 is therefore principally used to identify patients who should undergo a second-line test; it is not, by itself, diagnostic of significant or advanced fibrosis [8,9,10]. Single and repeated FIB-4 measurements may nevertheless help stratify long-term risk [11,12,13].

In PWH, obesity, insulin resistance, and chronic inflammation are associated with liver fibrosis through dysfunctional adipose tissue [14]. Obesity-associated insulin resistance promotes hepatic lipid accumulation, while cross-talk between adipocytes and macrophages amplifies cytokine release from adipose tissue [14]. The resulting lipotoxicity and low-grade inflammation activate hepatic stellate cells [3,6]. HIV infects adipose T cells early in infection, and the tissue in turn contributes to chronic immune activation [14]. A cross-sectional study of 405 HIV-monoinfected patients without other identified causes of liver disease found obesity and insulin resistance independently associated with advanced fibrosis after adjustment for metabolic syndrome, whereas HIV parameters and ART were not [15]. In PWH with MASLD, such single-occasion measurements cannot show whether change in weight and metabolic status over time is associated with change in FIB-4 independently of baseline status.

Immune status, HIV-related systemic inflammation, ART exposure, and concurrent metabolic changes are further considerations when FIB-4 is interpreted in PWH [16,17,18,19,20]. Mendeni et al. described longitudinal evolution of the aspartate aminotransferase-to-platelet ratio index and FIB-4 in PWH without viral hepatitis [21], while Guaraldi et al. reported associations of weight gain and MASLD with fibrosis-marker progression and regression in PWH over a longer follow-up [22]. Similar associations between MASLD and liver fibrosis have been reported in a Brazilian cohort of PWH [23], while factors associated with an elevated FIB-4 index have been described among HIV-monoinfected patients in Morocco [24]. The present study adds data from a MASLD-defined HIV cohort with frequent routine laboratory assessments and paired information on body weight and metabolic changes. It does not include an HIV-negative comparator and therefore was not designed to estimate an independent effect of HIV infection.

We aimed to describe short-term longitudinal changes in FIB-4 and metabolic parameters among PWH with MASLD and to identify factors associated with meeting a prespecified definition of FIB-4 increase.

## 2. Materials and Methods

### 2.1. Study Design and Participants

We conducted a retrospective longitudinal cohort study in the outpatient clinic of the Department of Infection and Immunity, Shanghai Public Health Clinical Center, Fudan University. Consecutive adults with documented HIV infection, aged 18–70 years, were screened from 25 March 2024 to 10 December 2024. At baseline, clinical history, abdominal ultrasonography, body weight, waist circumference, and blood pressure were recorded. Routine liver and kidney function tests and complete blood counts were obtained during outpatient follow-up, generally every three months. The study was conducted in accordance with the Declaration of Helsinki and was approved by the Ethics Committee of Shanghai Public Health Clinical Center (protocol code 2025-S006-01). Written informed consent was obtained from all participants.

### 2.2. Clinical and Laboratory Variables

Complete blood counts were measured using a Sysmex XN-1000 automated hematology analyzer (Sysmex Corporation, Kobe, Japan). HIV-related variables included HIV RNA, CD4+ and CD8+ T-cell counts, duration of HIV diagnosis, duration of ART, and ART regimen. Metabolic and biochemical variables included fasting glucose, fasting C-peptide, fasting insulin, glycated hemoglobin A1c, total cholesterol, triglycerides, ALT, AST, gamma-glutamyl transferase, height, weight, waist circumference, and blood pressure. Demographic data, comorbidities, medication exposure, and smoking and alcohol-use information were obtained from medical records.

### 2.3. Definition of MASLD

MASLD was defined according to the 2024 Chinese guideline [25]. Hepatic steatosis was identified by abdominal ultrasonography. MASLD required at least one cardiometabolic risk factor and exclusion of excessive alcohol consumption (≥210 g/week in men or ≥140 g/week in women) and other documented causes of hepatic steatosis or chronic liver disease.

### 2.4. FIB-4 Calculation and Study Outcome

FIB-4 was calculated as age (years) × AST (IU/L)/[platelet count (10^9^/L) × square root of ALT (IU/L)]. FIB-4 was calculated from available routine assessments. The primary classification compared baseline with the last available FIB-4 value. Among participants with baseline FIB-4 < 1.3, FIB-4 increase required both a relative increase of ≥20% and a last value of ≥1.3. Among participants with baseline FIB-4 ≥ 1.3, FIB-4 increase required a relative increase of ≥20% [13]. All other participants were classified as having no FIB-4 increase.

### 2.5. Statistical Analysis

Normally distributed continuous variables are presented as mean ± standard deviation and non-normally distributed variables as median (interquartile range). Categorical variables are presented as number (percentage). Between-group comparisons used the independent-samples *t*-test, Mann–Whitney U test, or chi-square test, as appropriate. Exploratory Cox proportional hazards models were used to evaluate factors associated with meeting the FIB-4 increase definition over the observed follow-up; variables with *p* < 0.05 in univariable analysis were then entered into a multivariable model. Logistic regression was used for obesity-status transition, and Spearman correlation was used to evaluate associations between weight change and changes in metabolic biomarkers. Tests were two-sided, with *p* < 0.05 considered statistically significant. Analyses were performed using R version 4.5.2 (R Foundation for Statistical Computing, Vienna, Austria), with the survival, ggalluvial, corrplot, and ggdist packages.

## 3. Results

### 3.1. Participant Characteristics

**Figure 1:**
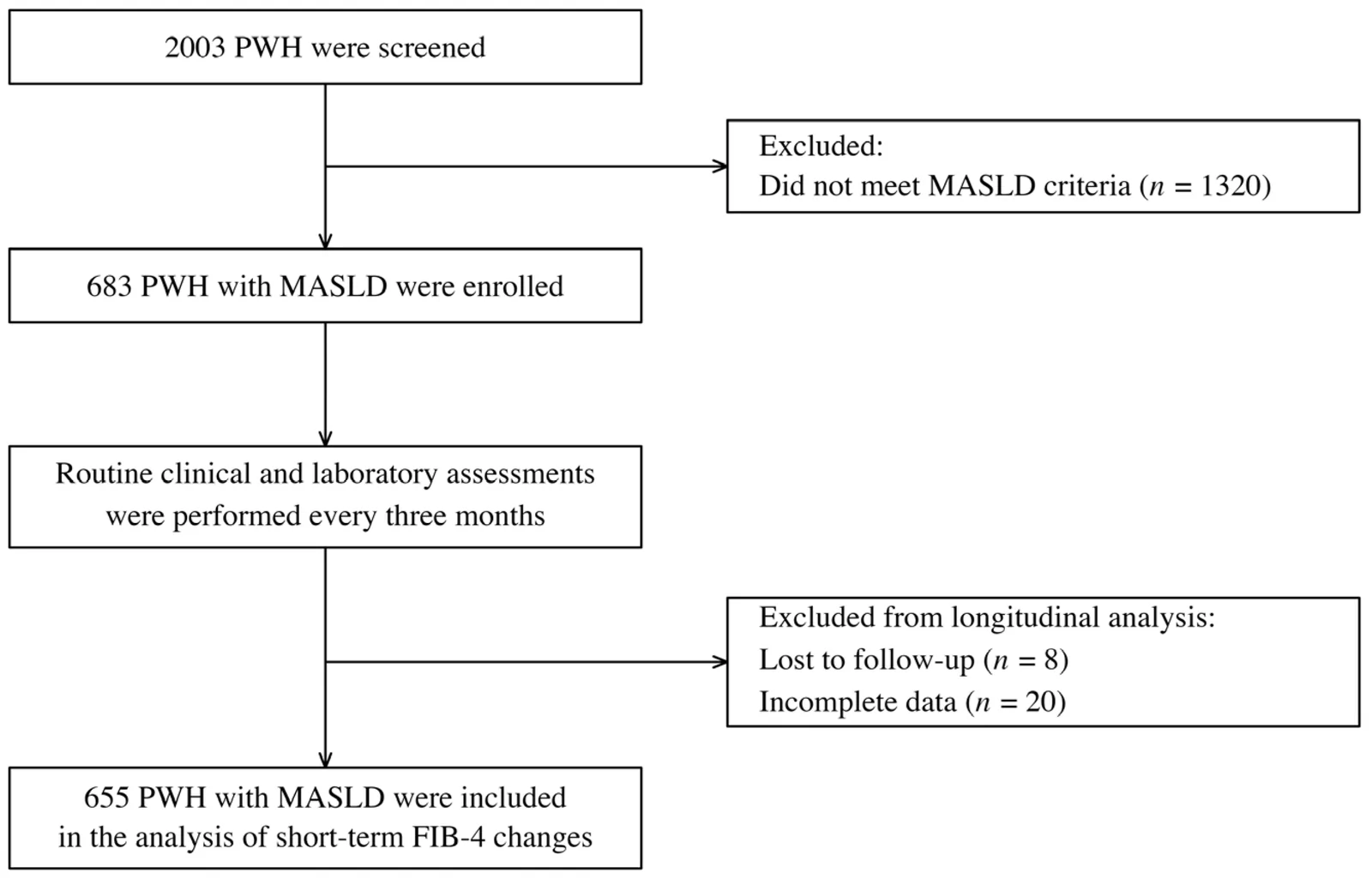
Flowchart of participant selection and longitudinal analysis. PWH, people with HIV; MASLD, metabolic dysfunction-associated steatotic liver disease; FIB-4, Fibrosis-4 index.

Of 2003 PWH screened, 1320 did not meet the MASLD criteria, and 683 were enrolled. Eight participants were lost to follow-up, and 20 had incomplete data, leaving 655 participants for longitudinal analysis (Figure 1). Their median age was 58 years, and 622 of the 683 enrolled participants (91.1%) were men. Baseline characteristics of participants included in the longitudinal analysis are shown in Table 1. Participants who later met the FIB-4 increase definition were older (median 61 versus 57 years, *p* < 0.001) and more frequently had hypertension (49.2% versus 30.2%, *p* = 0.003) and type 2 diabetes (25.4% versus 14.8%, *p* = 0.03) at baseline. Baseline body mass index, insulin resistance, liver enzymes, CD4 count, and antiretroviral regimen did not differ significantly between the groups.

### 3.2. Short-Term FIB-4 Increase and Associated Factors

After a median follow-up of 12.0 months (interquartile range 11.7–13.3), 59 of 655 participants (9.0%) met the prespecified FIB-4 increase definition and 596 (91.0%) did not, corresponding to 8.8 events per 100 person-years. In univariable Cox regression, baseline age (HR 1.08, 95% CI 1.04–1.11), hypertension (2.09, 1.25–3.50), type 2 diabetes (1.81, 1.00–3.25), and percentage weight change (1.20, 1.16–1.25) were associated with the outcome, whereas HIV- and antiretroviral therapy-related variables showed no association (Table 2). In multivariable analysis, percentage weight change (adjusted HR 1.19 per 1% increase, 95% CI 1.14–1.24; *p* < 0.001) and baseline age (adjusted HR 1.05, 95% CI 1.01–1.09; *p* = 0.006) remained associated with FIB-4 increase, whereas hypertension (adjusted HR 1.38, 95% CI 0.79–2.42; *p* = 0.25) and type 2 diabetes (adjusted HR 1.32, 95% CI 0.70–2.47; *p* = 0.39) did not.

### 3.3. Changes in Weight Category

**Figure 2:**
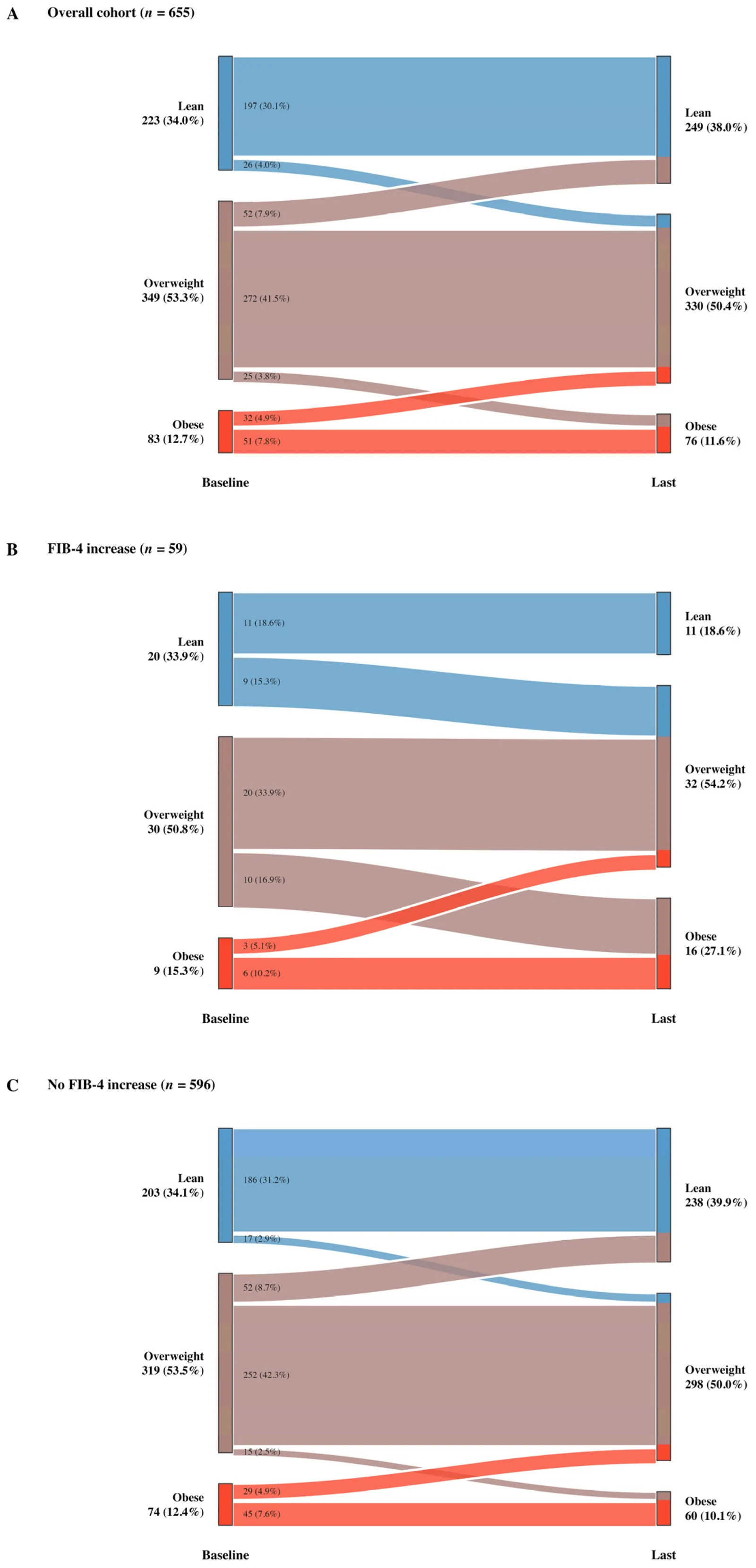
Weight-category transitions from baseline to the last follow-up in (**A**) the overall cohort, (**B**) participants with FIB-4 increase, and (**C**) participants without FIB-4 increase. Values are numbers of participants (percentages of the panel total). Lean, body mass index (BMI) < 24 kg/m^2^; overweight, BMI 24 to <28 kg/m^2^; obese, BMI ≥ 28 kg/m^2^.

Among the 655 participants, 223 (34.0%) were lean, 349 (53.3%) were overweight, and 83 (12.7%) were obese at baseline. At the last follow-up, 249 (38.0%) were lean, 330 (50.4%) were overweight, and 76 (11.6%) were obese. The overall distribution of BMI categories differed significantly between baseline and the last follow-up (*p* = 0.0085), with an overall shift toward lower BMI categories. In the FIB-4 increase group (*n* = 59), 10 participants (16.9%) transitioned from non-obesity to obesity. In contrast, in the no-increase group (*n* = 596), only 15 participants (2.5%) underwent the same transition. Movement to a higher weight category showed the same pattern: 19 participants (32.2%) in the FIB-4 increase group moved up, compared with only 3 (5.1%) who moved down. In the no-increase group, the direction was reversed, with 32 participants (5.4%) moving up and 81 (13.6%) moving down. Sankey diagrams show the weight-category transitions in the overall cohort and by FIB-4 change status (Figure 2).

Transition from non-obesity to obesity was associated with an increase in FIB-4 (OR 8.45, 95% CI 3.57–20.02; *p* < 0.001), whereas persistent obesity and transition from obesity to non-obesity were not statistically significant (Table 3).

### 3.4. Weight Change and Metabolic Biomarkers

**Figure 3:**
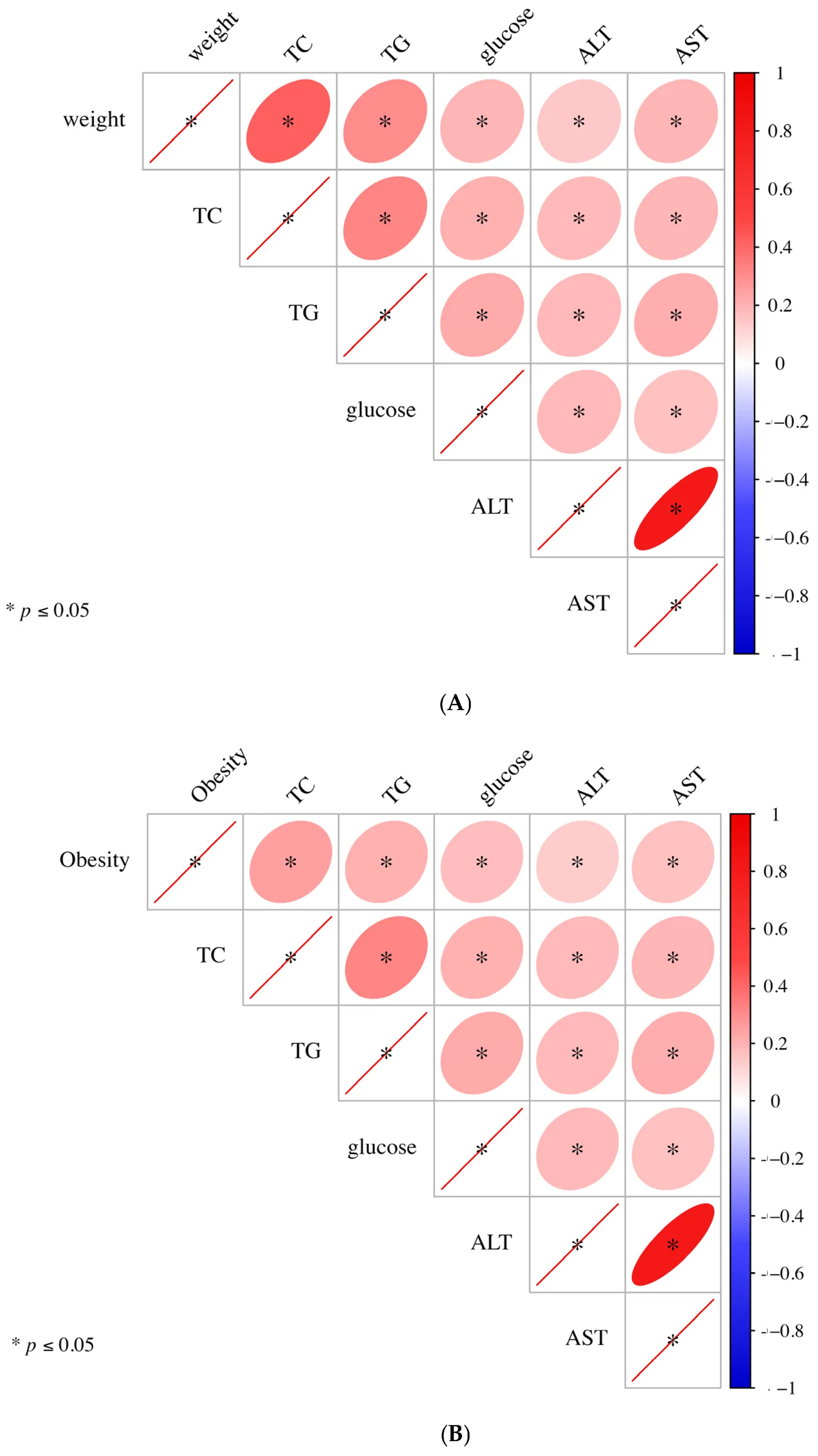
Correlations of (**A**) percentage weight change and (**B**) transition from non-obesity to obesity with changes in liver enzymes, fasting glucose, and lipid measures. Red indicates a positive Spearman correlation and blue indicates a negative correlation. Asterisks indicate *p* ≤ 0.05. TC, total cholesterol; TG, triglycerides; ALT, alanine aminotransferase; AST, aspartate aminotransferase.

Percentage weight change was positively correlated with changes in ALT (r = 0.15; *p* < 0.01), AST (r = 0.19; *p* < 0.01), fasting glucose (r = 0.19; *p* < 0.01), total cholesterol (r = 0.43; *p* < 0.01), and triglycerides (r = 0.30; *p* < 0.01). Transition from non-obesity to obesity was also correlated with changes in ALT (r = 0.14), AST (r = 0.17), fasting glucose (r = 0.17), total cholesterol (r = 0.25), and triglycerides (r = 0.20); all *p* values were <0.01 (Figure 3). Biomarker distributions by FIB-4 change status are shown in Figure S1.

## 4. Discussion

In this retrospective cohort of PWH with MASLD, 9.0% met a prespecified definition of short-term FIB-4 increase over approximately one year. Weight gain was independently associated with this outcome. Weight change was correlated with concurrent changes in lipids, fasting glucose and liver enzymes. The present cohort adds a MASLD-defined HIV population in which weight and metabolic measures were recorded in pairs over a short interval, so that change could be examined alongside baseline status. These results support attention to dynamic weight and metabolic change in PWH with MASLD.

Mendeni et al. followed 1112 PWH without hepatitis B or C after they started antiretroviral therapy and, over a mean of 2249 days of follow-up, recorded transition to a higher FIB-4 class at 6.4 per 100 person-years (95% CI 5.6–7.2) [21]. Guaraldi et al. followed 1183 PWH at three centers, of whom 25.2% had viral hepatitis coinfection (3.6% hepatitis B and 21.6% hepatitis C) and 46.8% had MASLD at baseline; over a median of 2.5 years, the cumulative incidence of liver stiffness-defined fibrosis progression was 8% (95% CI 6.5–9.9), and weight gain independently predicted progression (adjusted hazard ratio 3.12, 95% CI 1.41–6.90) [22]. In MASLD without HIV infection, Zhou et al. observed liver stiffness progression in 6.0% of 10,203 individuals over a median interval of 14.2 months [13]. Two cross-sectional studies in PWH are also relevant: Fittipaldi et al. found MASLD associated with clinically significant fibrosis in a Brazilian cohort [23], and Tahiri et al. identified factors associated with an elevated FIB-4 index among 619 HIV-monoinfected patients in Morocco [24]. In the present study, in which all participants had MASLD, 9.0% met the FIB-4 increase definition over a median follow-up of approximately one year, corresponding to 8.8 events per 100 person-years. In this cohort, baseline obesity and baseline insulin resistance were not associated with the outcome, whereas weight gain and transition from non-obesity to obesity were. Adiposity has more often been assessed on a single occasion: in 405 HIV-monoinfected patients, Lemoine et al. reported obesity (odds ratio 3.0, 95% CI 1.1–8.4) and the homeostasis model assessment index (1.1, 95% CI 1.007–1.2) as independent factors for advanced fibrosis after adjustment for the metabolic syndrome [15]. Weight change was tracked not only with liver enzymes but with the wider metabolic profile: the strongest correlation was with total cholesterol (r = 0.43), followed by triglycerides (0.30), while associations with fasting glucose and transaminases were weaker (0.15–0.19). Short-term weight change therefore marks a coordinated metabolic shift.

FIB-4 is useful as an initial risk-stratification test, especially where elastography is not immediately available, but its components create important interpretive limitations. AST and ALT may change with weight, steatosis, medication exposure, intercurrent illness, and other transient conditions. Platelet count may also vary for reasons unrelated to hepatic fibrosis. Confirmation with vibration-controlled transient elastography, magnetic resonance elastography, the Enhanced Liver Fibrosis test, or other validated second-line tests would be required before interpreting these changes as fibrosis. Whether a change in this size exceeds ordinary variation in the component tests remains a fair question. The same definition has been tested against histology: in a paired liver biopsy cohort, Zhou et al. found progression in 35.4% of participants whose low baseline FIB-4 rose and 49.4% of those whose high baseline FIB-4 rose, against 17.5% of those low and stable (adjusted odds ratios 2.20 and 3.68), with regression showing the mirror pattern (0.36 and 0.22) [13]. The same definition, applied roughly a year after baseline, predicted liver-related events over a median of 56.1 months in 41,105 individuals (adjusted hazard ratios of 2.55 with a low and 6.85 with a high baseline FIB-4), and was also associated with all-cause death and cardiovascular events [13]. In the general population, a one-unit rise over a mean interval of 2.4 years carried a comparable signal [12].

Baseline age was associated with the FIB-4 increase outcome, but age is included in the FIB-4 equation. This creates mathematical coupling and precludes an etiological interpretation of age as an independent cause of the observed index change. Similarly, the threshold of 1.3 should be understood as a triage threshold for additional assessment rather than a diagnostic threshold for significant fibrosis [8,9,10].

PWH may have additional determinants of liver and metabolic health, including chronic immune activation and HIV-related inflammation [16,17,26,27,28], changes in adipose tissue associated with HIV infection and its treatment [14], and longitudinal changes in non-invasive fibrosis indices during antiretroviral therapy [18,29,30]. We did not identify clear associations between HIV- or ART-related variables and short-term increases in FIB-4. The study had no HIV-negative comparison group, and HIV infection should therefore be viewed as the clinical context of the cohort rather than an effect estimated by the present analysis.

This study has several limitations. First, FIB-4 is an indirect marker, and the approximately one-year follow-up is too short to establish meaningful histological fibrosis progression in MASLD. Second, the primary classification used baseline and last available values and may be affected by regression to the mean and transient laboratory fluctuations. Third, no elastography, Enhanced Liver Fibrosis testing, magnetic resonance-based assessment, or liver histology was available for confirmation. Fourth, the retrospective single-center design limited standardization of ultrasound operators, machine calibration, alcohol assessment, and retention procedures, and may have introduced residual confounding and selection bias. Fifth, only 59 participants met the outcome definition, limiting the complexity and precision of multivariable models. Finally, genetic factors such as patatin-like phospholipase domain-containing protein 3 (PNPLA3) were not assessed [31]. Longer follow-up with repeated continuous FIB-4 modeling and confirmatory liver-stiffness or biomarker measurements is needed.

## 5. Conclusions

Among PWH with MASLD, weight gain was associated with meeting a short-term FIB-4 increase definition and with concurrent changes in lipids, fasting glucose and liver enzymes. The findings support monitoring weight and metabolic health and using elevated or increasing FIB-4 to prompt confirmatory liver assessment.

## Figures and Tables

**Table 1:** Baseline characteristics according to short-term FIB-4 change status.

Characteristics	FIB-4 Increase (*n* = 59)	No FIB-4 Increase (*n* = 596)	*p* Value
Age (years)	61 (59–68)	57 (50–63)	<0.001
Male sex, *n* (%)	51 (86.4)	546 (91.6)	0.18
Follow-up, months	12.2 (11.6–13.3)	12 (11.7–13.3)	0.86
BMI (kg/m^2^)	24.9 (23.4–26.7)	24.9 (23.2–26.7)	0.76
Hypertension, *n* (%)	29 (49.2%)	180 (30.2%)	0.003
Duration of ART (days)	2240 (1108–4058)	2604 (1266–3975)	0.44
Duration of diagnosis of HIV (days)	2286 (1144–4068)	2749 (1286–4257)	0.44
Obesity (BMI ≥ 28), *n* (%)	9 (15.3%)	74 (12.4%)	0.53
Central obesity (waist), *n* (%)	31 (52.5%)	315 (52.9%)	0.964
T2D, *n* (%)	15 (25.4%)	88 (14.8%)	0.03
IR, *n* (%)	43 (72.9%)	476 (79.9%)	0.21
Smoke use, *n* (%)	14 (23.7%)	168 (28.2%)	0.47
Alcohol use, *n* (%)	7 (11.9%)	74 (12.4%)	0.90
NNRTI use, *n* (%)	28 (47.5%)	303 (50.8%)	0.62
NRTI use, *n* (%)	59 (100%)	590 (99.0%)	1.00
PI use, *n* (%)	0 (0%)	14 (2.3%)	0.63
INSTI use, *n* (%)	31 (52.5%)	274 (46.0%)	0.34
AZT use, *n* (%)	31 (52.5%)	254 (42.6%)	0.14
EFV use, *n* (%)	25 (42.4%)	274 (46.0%)	0.60
Immunorecognition insufficiency, *n* (%)	17 (28.8%)	128 (21.5%)	0.20
CD4, counts	490 (335–702)	521 (366–719)	0.66
Ratio of CD4 to CD8	0.7 (0.4–1.0)	0.7 (0.4–1.0)	0.44
Fasting glucose (mmol/L)	6.2 (5.6–7.6)	6.3 (5.7–7.5)	0.42
HbA1c (%)	6.0 (5.7–6.4)	6.0 (5.6–6.5)	0.83
ALT (IU/L)	25.8 (18.3–36.9)	26.9 (19.8–40.2)	0.35
AST (IU/L)	25.1 (19.5–32)	23.2 (19.0–30.1)	0.49
GGT (IU/L)	34 (26–63)	36 (26–60)	0.99
Fasting insulin	19.7 (10.5–44.2)	18.7 (11.5–36.0)	0.80
Fasting C-peptide	4.9 (3.1–8.3)	4.2 (2.8–6.7)	0.06
HOMA-IR	5.3 (2.6–14.2)	5.58 (3.03–12.48)	0.93
ACEI/ARB use, *n* (%)	8 (13.6%)	55 (9.2%)	0.28
SGLT2i use, *n* (%)	2 (3.4%)	6 (1.0%)	0.16
GLP-1RA use, *n* (%)	1 (1.7%)	1 (0.2%)	0.17

FIB-4, Fibrosis-4 index; HIV, human immunodeficiency virus; BMI, body mass index; T2D, type 2 diabetes; IR, insulin resistance; ART, antiretroviral therapy; NNRTI, non-nucleoside reverse transcriptase inhibitor; NRTI, nucleoside reverse transcriptase inhibitor; PI, protease inhibitor; INSTI, integrase strand transfer inhibitor; AZT, zidovudine; EFV, efavirenz; ALT, alanine aminotransferase; AST, aspartate aminotransferase; GGT, gamma-glutamyl transferase; HbA1c, glycated hemoglobin A1c; CD4, cluster of differentiation 4; CD8, cluster of differentiation 8; HOMA-IR, homeostasis model assessment of insulin resistance; ACEI, angiotensin-converting enzyme inhibitor; ARB, angiotensin II receptor blocker; SGLT2i, sodium-glucose cotransporter 2 inhibitor; GLP-1RA, glucagon-like peptide-1 receptor agonist.

**Table 2:** Exploratory Cox regression analysis of factors associated with short-term increases in FIB-4.

Predictors	HR	*p* Value	aHR	*p* Value
Age (years)	1.08 (1.04–1.11)	<0.001	1.05 (1.01–1.09)	0.006
Male, *n* (%)	0.55 (0.26–1.17)	0.12		
BMI (kg/m^2^)	1.01 (0.92–1.11)	0.82		
Obesity, *n* (%)	1.05 (0.51–2.14)	0.89		
T2D, *n* (%)	1.81 (1.00–3.25)	0.049	1.32 (0.70–2.47)	0.39
IR, *n* (%)	0.74 (0.40–1.35)	0.33		
Hypertension, *n* (%)	2.09 (1.25–3.50)	0.01	1.38 (0.79–2.42)	0.25
Duration of ART (days)	1.00 (1.00–1.00)	0.59		
Duration of diagnosis of HIV (days)	1.00 (1.00–1.00)	0.54		
Smoke use, *n* (%)	0.82 (0.45–1.49)	0.51		
Alcohol use, *n* (%)	0.86 (0.39–1.90)	0.71		
NNRTI use, *n* (%)	0.88 (0.52–1.48)	0.63		
INSTI use, *n* (%)	1.26 (0.75–2.12)	0.38		
AZT use, *n* (%)	1.55 (0.92–2.59)	0.10		
EFV use, *n* (%)	0.87 (0.52–1.47)	0.61		
Immunorecognition insufficiency, *n* (%)	1.23 (0.69–2.20)	0.47		
CD4, counts	1.00 (1.00–1.00)	0.18		
Ratio of CD4 to CD8	1.01 (0.66–1.56)	0.95		
Fasting glucose (mmol/L)	0.96 (0.86–1.07)	0.47		
HbA1c (%)	0.84 (0.62–1.13)	0.25		
ALT (IU/L)	1.00 (0.98–1.01)	0.73		
AST (IU/L)	0.99 (0.98–1.01)	0.38		
GGT (IU/L)	1.00 (0.99–1.01)	0.97		
Fasting insulin	1.00 (1.00–1.01)	0.68		
Fasting C-peptide	1.04 (0.99–1.09)	0.12		
HOMA-IR	1.00 (0.98–1.02)	0.99		
Weight increase (%)	1.20 (1.16–1.25)	<0.001	1.19 (1.14–1.24)	<0.001

HR and *p* values are from univariable Cox regression (one predictor per model). The multivariable Cox model included the four variables associated with the outcome in univariable analysis (baseline age, hypertension, type 2 diabetes, and percentage weight change) and was fitted in 651 participants with complete data on these variables (59 outcome events). HR, hazard ratio; aHR, adjusted hazard ratio; CI, confidence interval; FIB-4, Fibrosis-4 index; HIV, human immunodeficiency virus; BMI, body mass index; T2D, type 2 diabetes; IR, insulin resistance; ART, antiretroviral therapy; NNRTI, non-nucleoside reverse transcriptase inhibitor; INSTI, integrase strand transfer inhibitor; AZT, zidovudine; EFV, efavirenz; CD4, cluster of differentiation 4; CD8, cluster of differentiation 8; HbA1c, glycated hemoglobin A1c; ALT, alanine aminotransferase; AST, aspartate aminotransferase; GGT, gamma-glutamyl transferase; HOMA-IR, homeostasis model assessment of insulin resistance.

**Table 3:** Association between obesity status transition and short-term FIB-4 increase.

Change in Obesity Status	OR (95% CI)	*p* Value
Persistent non-obesity	Ref	
Non-obesity to obesity	8.45 (3.57–20.02)	<0.001
Persistent obesity	1.69 (0.68–4.20)	0.259
Obesity to non-obesity	1.31 (0.38–4.49)	0.666

OR, odds ratio; CI, confidence interval.

## Data Availability

De-identified data supporting the findings of this study are available from the corresponding author upon reasonable request, subject to institutional ethics approval and participant confidentiality requirements.

## Supplementary Materials

The following supporting information can be downloaded at: https://www.mdpi.com/article/10.3390/v18080896/s1, Figure S1: Changes in body weight, fasting glucose, liver enzymes, and lipid measures according to short-term FIB-4 change status.

## Abbreviations

The following abbreviations are used in this manuscript:

ALT	Alanine aminotransferase
ART	Antiretroviral therapy
AST	Aspartate aminotransferase
BMI	Body mass index
FIB-4	Fibrosis-4 index
HOMA-IR	Homeostasis model assessment of insulin resistance
MASLD	Metabolic dysfunction-associated steatotic liver disease
PWH	People with HIV
