# Supplementary material for: Weight Gain Is Associated with Short-Term FIB-4 Increase and Concurrent Metabolic Change in People with HIV and Metabolic Dysfunction-Associated Steatotic Liver Disease
Source: Viruses. 2026 Aug 14;18(8):896. doi: 10.3390/v18080896 (PMC13517362; doi:10.3390/v18080896)

Figure S1

Changes in body weight, fasting glucose, liver enzymes, and lipid measures according to short-term FIB-4 change status. FIB-4, Fibrosis-4 index; TC, total cholesterol; TG, triglycerides; ALT, alanine aminotransferase; AST, aspartate aminotransferase. Groups are labeled according to short-term FIB-4 change status (FIB-4 increase vs no FIB-4 increase) at baseline and last follow-up. (A) Body weight; (B) Fasting glucose; (C) ALT; (D) AST; (E) Total cholesterol; (F) Triglycerides.

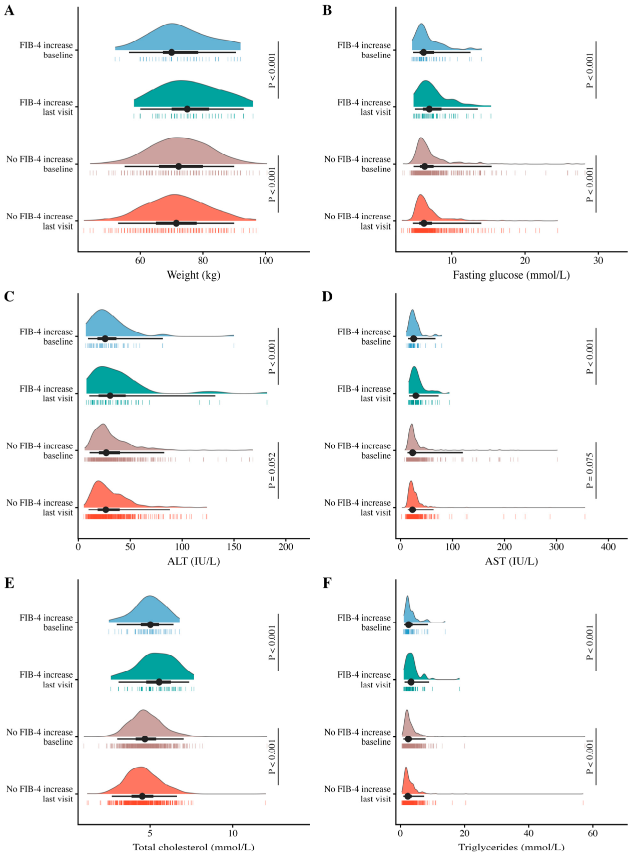

Supplement: Supplementary file 1 [file viruses-18-00896-s001.zip › viruses-4448746-supplementary.pdf]
